# Clinical neurophysiological assessment of sepsis-associated brain dysfunction: a systematic review

**DOI:** 10.1186/s13054-014-0674-y

**Published:** 2014-12-08

**Authors:** Koji Hosokawa, Nicolas Gaspard, Fuhong Su, Mauro Oddo, Jean-Louis Vincent, Fabio Silvio Taccone

**Affiliations:** Department of Intensive Care, Erasme University Hospital, Université Libre de Bruxelles, Route de Lennik 808, 1070 Brussels, Belgium; Comprehensive Epilepsy Center and Computational Neurophysiology Laboratory, Dept. of Neurology, School of Medicine, Yale University, Yale-New Haven Hospital, New Haven, CT 06520 USA; Department of Neurology, Erasme University Hospital, Université Libre de Bruxelles, Route de Lennik 808, 1070 Brussels, Belgium; Department of Intensive Care Medicine, Lausanne University Hospital (Centre Hospitalier Universitaire Vaudois), Rue du Bugnon 46, 1011 Lausanne, Switzerland

## Abstract

**Introduction:**

Several studies have reported the presence of electroencephalography (EEG) abnormalities or altered evoked potentials (EPs) during sepsis. However, the role of these tests in the diagnosis and prognostic assessment of sepsis-associated encephalopathy remains unclear.

**Methods:**

We performed a systematic search for studies evaluating EEG and/or EPs in adult (≥18 years) patients with sepsis-associated encephalopathy. The following outcomes were extracted: a) incidence of EEG/EP abnormalities; b) diagnosis of sepsis-associated delirium or encephalopathy with EEG/EP; c) outcome.

**Results:**

Among 1976 citations, 17 articles met the inclusion criteria. The incidence of EEG abnormalities during sepsis ranged from 12% to 100% for background abnormality and 6% to 12% for presence of triphasic waves. Two studies found that epileptiform discharges and electrographic seizures were more common in critically ill patients with than without sepsis. In one study, EEG background abnormalities were related to the presence and the severity of encephalopathy. Background slowing or suppression and the presence of triphasic waves were also associated with higher mortality. A few studies demonstrated that quantitative EEG analysis and EP could show significant differences in patients with sepsis compared to controls but their association with encephalopathy and outcome was not evaluated.

**Conclusions:**

Abnormalities in EEG and EPs are present in the majority of septic patients. There is some evidence to support EEG use in the detection and prognostication of sepsis-associated encephalopathy, but further clinical investigation is needed to confirm this suggestion.

## Introduction

Acute brain dysfunction, characterized by altered mental status, commonly occurs during sepsis and typically develops early [1,2], often before alterations in other organ function [3,4]. This syndrome has been referred to as sepsis-associated encephalopathy (SAE) [5] or, more recently, sepsis-associated brain dysfunction (SABD) [6] and overlaps with the syndrome of delirium associated with critical illness. The pathophysiology of SAE/SABD is multifactorial and presumably related to the effects of systemic inflammation on cerebral perfusion and neuronal activity, in the absence of direct infection of the central nervous system (CNS) [7,8]. Increased severity of this encephalopathy has been associated with worse outcome, especially in the setting of multiple organ failure [4-6,9,10].

Electroencephalography (EEG) measures spontaneous electrical activity generated by synaptic transmission in the superficial layers of the cerebral cortex and modulated by subcortical structures from the upper brainstem to the thalamus. The raw EEG can be inspected visually or analyzed using quantitative methods (quantitative EEG, qEEG) that extract descriptive features, such as frequency, amplitude, power, linearity. Evoked potentials (EPs) measure brain responses to sensory stimulation [11], including responses generated by subcortical structures (brainstem auditory evoked potentials (BAEPs); N14 and P18 somatosensory evoked potentials (SSEPs)), by thalamo-cortical input to the primary sensory cortices (N20 SSEP, middle latency AEPs) and by intrinsic cortical activity (N70 SSEP, mismatch negativity) [12]. EEG and EPs are objective tests that can demonstrate the presence and extent of brain dysfunction and may complement the clinical examination in specific populations of critically ill patients, for example following anoxic brain injury [12,13]. However, it remains unclear whether EEG or EPs has a potential role in the detection and quantification of SAE/SABD, and/or whether they provide any useful prognostic information.

The aim of this study was, therefore, to review the available clinical literature on the role of electrophysiological tests to diagnose SAE/SABD and to evaluate the impact of EEG or EP abnormalities on the outcome of patients with SAE/SABD.

## Methods

This systematic review was performed according to the Preferred Reporting Items for Systematic Reviews and Meta-Analyses (PRISMA) statement [14].

The aim of our study was to answer the following questions:

1. What is the incidence of EEG/EP alterations in patients with severe infections or sepsis?
2. What is the accuracy of EEG/EP abnormalities in the diagnosis of SAE/SABD?
3. What is the prognostic value of such abnormalities in this setting?

### Data collection 

A systematic review was conducted including articles published from 1 January 1966 to 31 December 2013 in the PubMed database, using the terms ‘infection’ OR ‘inflammation’ OR ‘sepsis’ OR ‘septic shock’ OR ‘severe sepsis’ OR ‘delirium’ OR ‘encephalopathy’ with: ‘electroencephalography’ OR ‘electroencephalogram’ OR ‘EEG monitoring’ OR ‘EEG’ OR ‘evoked potential.’ The reference lists of review articles were also checked for relevant studies. The search was restricted to English language articles.

One author (KH) reviewed the full-text articles to select eligible studies according to the PICO approach: 1) patient population, that is, patients suffering from systemic infection, sepsis; 2) intervention provided, that is, monitoring of EEG or EPs; 3) controls, that is, patients with infection or sepsis without SAE/SABD, or healthy individuals; 4) outcome endpoints, that is, incidence of EEG/EP abnormalities, diagnosis of SAE/SABD, ICU/hospital outcome.

Unpublished data from congress presentations or abstracts were not considered. Also excluded from the review were: 1) review articles; 2) case reports or case series with ≤5 patients; 3) animal or other experimental studies; 4) studies on pediatric populations (<18 years old); 5) studies that included only intracranial infections; and 6) studies on healthy volunteers (that is, receiving endotoxin). Duplicate publications of identical series were excluded (that is, only one was included).

Data were abstracted using a predefined abstraction spreadsheet, according to the PICO system. The following information was extracted from the studies that met inclusion criteria: study design and location, number of participants, patient inclusion criteria, rate of sepsis or infection, Acute Physiology and Chronic Health Evaluation (APACHE) II score, number of patients receiving drugs that may influence brain function (that is, sedatives or opiates), types of EEG, EEG and EP findings and survival rate.

EEG findings were classified into:1) background abnormalities, that is, background slowing, presence or absence of spontaneous background variability, presence or absence of reactivity, presence or absence of normal sleep transients, periods of background suppression including suppression-burst pattern; 2) periodic and rhythmic discharges, that is, triphasic waves (TWs), frontal intermittent rhythmic delta activity (FIRDA), general periodic epileptiform discharges (GPEDs), periodic lateralized epileptiform discharges (PLEDs) or bilateral independent lateralized epileptiform discharges (BIPLEDs); 3) ictal discharges, that is, electrographic seizures or status epilepticus [15]; 4) qEEG analysis; and 5) changes in EP. Diagnostic and predictive values for different outcomes were also collected or calculated if not provided by the authors. No attempt was made to re-analyse the data or to obtain additional unpublished data from the authors of the selected publications. The accuracy of data extraction was controlled by two co-authors (NG, FST).

## Results

**Figure 1:**
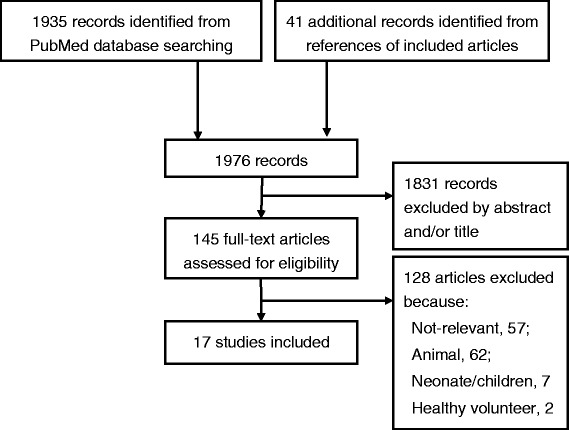
**Preferred reporting items for systematic reviews and meta-analyses (PRISMA) flow diagram showing selection processes.**

Among 1,976 citations, 145 articles were suitable for full-text reviewing, and 17 studies were eventually selected (Figure 1). These 17 studies included:

- one case-control study, which compared EEG findings in 43 patients with systemic infection and 30 controls [16];
- seven case series of patients with sepsis (total number of patients, n = 272) [17-23];
- one case-control study of patients with GPEDs, which included patients with sepsis [24];
- eight case series that included patients with and without sepsis (total n = 1,360 patients, 49% with sepsis) [25-32].

The methodological characteristics of the studies are shown in Table 1. Five studies were retrospective [24,27,28,30,32], eight prospective [16-18,21,25,26,29,31] and the study design was not mentioned in the four other studies [19,20,22,23].

**Table 1:** **Summary of included studies**

	**Ref**	**Study design**	**Location (ICU patients,%)**	**Number**	**Inclusion criteria**	**Patients with sepsis/infections (number, %)**	**APACHE II score**	**Survival (number, %)**	**Patients on sedatives and/or opiates (number, %)**	**Topic**	**Time to EEG/EPs (days)**	**EEG/EPs as primary outcome?**
**Case-control study (patients with systemic infection versus controls)**												
	16	P	Internal medicine (NA)	73	CAP	43 (59%)	13 ± 4	NA	NA	qEEG VEP	1	Y
**Case series of patients with sepsis**												
	21	P	Medical and surgical ICU (100%)	71	SS with brain dysfunction^a^	71 (100%)	NA (SAPS II 49 (38 to 60))	48 (68%)	49 (69%)	EEG	NA	N
	20	NA	ICU (100%)	30	SS^a^	30 (100%)	32 ± 6	NA	30 (100%)	EEG	4	N
	17	P	Medical ICU (100%)	68	SS	68 (100%)	NA (APACHE III, 101 ± 26)	31 (46%)	33 (49%)	SSEP	1 to 2	Y
	19	NA	ICU (100%)	14	Sepsis	14 (100%)	18.6 ± 6.0	8 (57%)	12 (85%)	qEEG	1 to 5	Y
	18	P	ICU (100%)	69	Severe Infections^b^	69 (100%)	NA	46 (67%)	0	EEG	NA	Y
	22	NA	ICU(100%)	6	Severe Infections^b^	6 (100%)	NA	1 (17%)	0	EEG AEP	1 to 11	N
	23	NA	NA (NA)	14	Severe Infections^b^	14 (100%)	NA	14 (100%)	NA	EEG	NA	Y
**Case series or case-control studies including patients with and without sepsis**												
	32	R	Surgical ICU (100%)	154	Encephalopathy +/− seizures^a^	100 (65%)	NA	73 (47%)	106 (70%)	cEEG	4 to 9	Y
	28	R	Hospital (70%)	154	Encephalopathy	72 (47%)	NA	135 (88%)	20 (13%)	EEG	NA	Y
	27	R	Hospital (81%)	105	Encephalopathy and TWs	62 (59%)	NA	84 (80%)	20 (19%)	EEG	NA	Y
	24	R	Hospital (88%)	400	GPDs	144 (36%)	NA	249 (62%)	NA	cEEG	NA	Y
	30	R	Medical ICU (100%)	201	Encephalopathy +/− seizures	120 (60%)	NA	NA	29 (14%)	cEEG	NA	Y
	26	P	Adult ICU (100%)	125	Patients on MV^a^	78 (62%)	27.4 ± 8.2	93 (74%)	123 (98%)	qEEG (BIS)	NA	Y
	29	P	Surgical ICU (100%)	54	Post-abdominal surgery	24 (44%)	NA (SAPS II, 34 (22 to 48))	NA	0	AEP	1	Y
	31	P	Burns ICU (100%)	64	SIRS	44 (69%)	NA	NA	NA	EEG	NA	N
	25	P	Medical ICU (100%)	103	MOF and Encephalopathy	74 (72%)	NA (APACHE III, 101 ± 24)	52 (50%)	60 (58%)	SSEP	1 to 2	Y

^a^excluded pre-existing neurological or psychiatric diseases; ^b^excluded infections of the central nervous system. AEP, auditory evoked potentials; APACHE, Acute Physiology and Chronic Health Evaluation; BIS, bispectral index; CAP, community-acquired pneumonia; cEEG, continuous EEG monitoring; CI, confidence interval; CNS, central nervous system; EEG, electroencephalogram; GPDs, general periodic discharges; ICU, intensive care unit; MOF, multiple organ failure; MV, mechanical ventilation; N, No; NA, not available; P, prospective; qEEG, quantitative electroencephalography; R, retrospective; SAPS, Simplified Acute Physiology Score; SE, status epilepticus; SIRS, systemic inflammatory response syndrome; SS, severe sepsis or septic shock; SSEP, somatosensory evoked potential; TWs, triphasic waves; WBC, white blood cell; VEP, visual evoked potentials; Y, Yes.

The inclusion criteria were different in the various studies. Eleven studies used EEG, including eight with intermittent EEG [18,20-23,27,28,31] and three with continuous EEG monitoring [24,30,32]. Quantitative EEG was performed in three studies [16,19,26], including one study where the bispectral index (BIS) was monitored [26]. Five studies used EPs [16,17,22,25,29] and one used EEG and EPs [22]. The median time from sepsis admission to EEG/EPs recording varied from one to eleven days; however, in nine studies this information was not reported. Finally, in 4/17 reports, EEG/EPs findings were not the primary aim of the studies.

### Raw EEG signal findings 

Results from visual inspection of the raw EEG were available for five studies including only septic patients [18,20-23] and for six studies that examined mixed ICU populations [24,27,28,30-32], with a proportion of septic patients that ranged from 36% to 69% (Table 2).

**Table 2:** **The incidence and significance of background abnormalities, periodic and rhythmic discharges and electrographic seizures in sepsis**

	**Ref**	**Type of EEG abnormality**	**Details**	**Rate (number, %)**	**Diagnostic and prognostic values of the changes**
**Case series of patients with sepsis**					
	21	B	Slow waves; malignant EEG pattern	5/43 (12%); 15/43 (35%)	Malignant EEG pattern associated with chronic leukoencephalopathy and acute brain ischemia.
		PD/RD	TWs	5/43 (12%)	NA
		Esz	ESz	13/43 (30%)	NA
	20	B	Theta-delta; delta; burst-suppression	2/18 (11%); 12/18 (67%); 2/18 (11%)	NA
		PD/RD	TWs	1/18 (6%)	NA
	18	B	Theta; delta; suppression; no reactivity	16/62 (26%); 22/62 (35%); 8/62 (13%), 9/62 (15%)	Severity of SAE associated with severity of EEG abnormalities Delta (OR = 2.4) and suppression (OR = 4.5) associated with mortality.
		PD/RD	TWs	5/60 (10%)	TWs associated with mortality (OR = 1.5)
	22	B	Theta; delta	4/6 (67%); 2/6 (33%)	NA
	23	B	Slow wave sleep; Theta-delta; delta; Paroxysmal theta	4/14 (29%); 2/14 (14%); 1/14(7%); 1/14 (7%)	NA
		PD/RD	Paroxysmal slowing with sharp waves	2/14 (14%)	NA
**Case series or case-control studies that included patients with and without sepsis**					
	32	B	No reactivity	28/152 (18%)	No reactivity tended to be associated with poor outcome (OR = 2.8, *P* = 0.13)
		PD/RD	PEDs (including GPDs, PLEDs and BIPLEDs)	45 (29 %)	PEDs persisting for >24 h associated with poor outcome (OR = 2.9, *P* = 0.01)
		Esz	NCSz (including NCSE)	24/154 (16 %) (8/154 (5 %))	NCSz associated with poor outcome (OR = 10.4, *P* = 0.04)
	27	B	Predominant theta; theta/delta; no reactivity	30/105 (29%); 55/105 (52%); 22/105(21%)	Lack of EEG background reactivity associated with mortality (OR = 3.7, *P* = 0.04)
		PD/RD	TWs	105/105 (100%)	NA
	28	B	Theta; theta/delta; delta; FIRDA	34/154 (22%); 32/154 (21%); 28/154 (18%); 26/154 (18%)	Theta/delta and delta associated with more severe alteration of consciousness; Theta/delta associated with poor outcome (OR = 2.5, *P* = 0.03); FIRDA associated with good outcome (OR = 4.8, p = 0.004)
		PD/RD	TWs	34/154 (22%)	TWs associated with more severe alteration of consciousness and with higher mortality (OR = 4.5, *P* = 0.005)
	24	PD/RD	GPDs including TWs;	(50%, case-matched cohort); 63/400 (16%); 24/400 (6%)	No significant difference in poor outcome between GPDs and controls; GPDs associated with a longer ICU stay (18 days versus 15 days, *P* = 0.002)
		Esz	(Seizures)	(73/400 (18%))	NA
	30	PD/RD	PEDs (including GPDs, PLEDs and BIPLEDs)	34/201 (17%);	Diagnostic value of the presence of ESz or PEDs for sepsis (Sen = 0.32; Spe = 0.91; PLR = 3.7; NLR = 0.7) ESz or PEDs associated with mortality or severe disability at hospital discharge (adjusted OR = 19.1, *P* = 0.001).
		Esz	ESz	21/201 (10%)	NA
	31	B	Slow waves	27/27 (100%)	NA
		PD/RD	TWs	1/27 (4%)	NA

B, background; EEG, electroencephalogram; ESz, electrographic seizures; FIRDA, frontal intermittent delta activity; GPDs, general periodic discharges; NA, not applicable; NCSE, nonconvulsive status epilepticus; NCSz, nonconvulsive seizures; NLR, negative likelihood ratio; OR, odds ratio; (P)(BI)LEDs, (periodic) (bilateral independent) lateralized epileptiform discharges; PD/RD: periodic and rhythmic discharges; PEDs, periodic epileptiform discharges; PLR, positive likelihood ratio; SAE, sepsis-associated encephalopathy; Sen, sensitivity; Spe, specificity; TWs, triphasic waves.

#### Background abnormalities

Five case series reported the prevalence of background abnormalities in septic patients [18,20-23]. A small prospective case series found an abnormal EEG background in 6/6 patients with SAE; however, the small sample size prevented the authors from studying the association of these abnormalities with the severity of SAE [22]. In a prospective series of 62 patients with sepsis, 54 (87%) had an abnormal EEG, consisting of background abnormalities that included continuous theta and delta slowing, or a suppression-burst pattern [18]. The presence and severity of EEG background abnormalities was associated with altered clinical findings consistent with a diagnosis of SAE as well as the severity of SAE and increased mortality, although 50% of the patients with no signs of encephalopathy also had an abnormal EEG. Reactivity was absent in 9/62 (15%) patients but did not correlate with outcome.

A similarly high incidence of background abnormalities was found in another study on septic patients, although the authors did not find any association between EEG findings and the presence of SAE [20]. In a more recent, prospective study of 43 septic patients with clinical evidence of SAE, 5 (12%) patients had ‘slow EEG waves’ and 15 (35%) had a ‘malignant’ EEG pattern (suppression-burst, continuous attenuation or suppression) [21]. The presence of a ‘malignant’ pattern was associated with the presence of acute ischemic injury on brain imaging; however, the association with the severity of SAE and outcome was not assessed. None of these studies used continuous EEG monitoring so that the persistence of these EEG abnormalities, especially through the nycthemeral cycle, and the presence of normal EEG transients of sleep, were not assessed.

**Figure 2:**
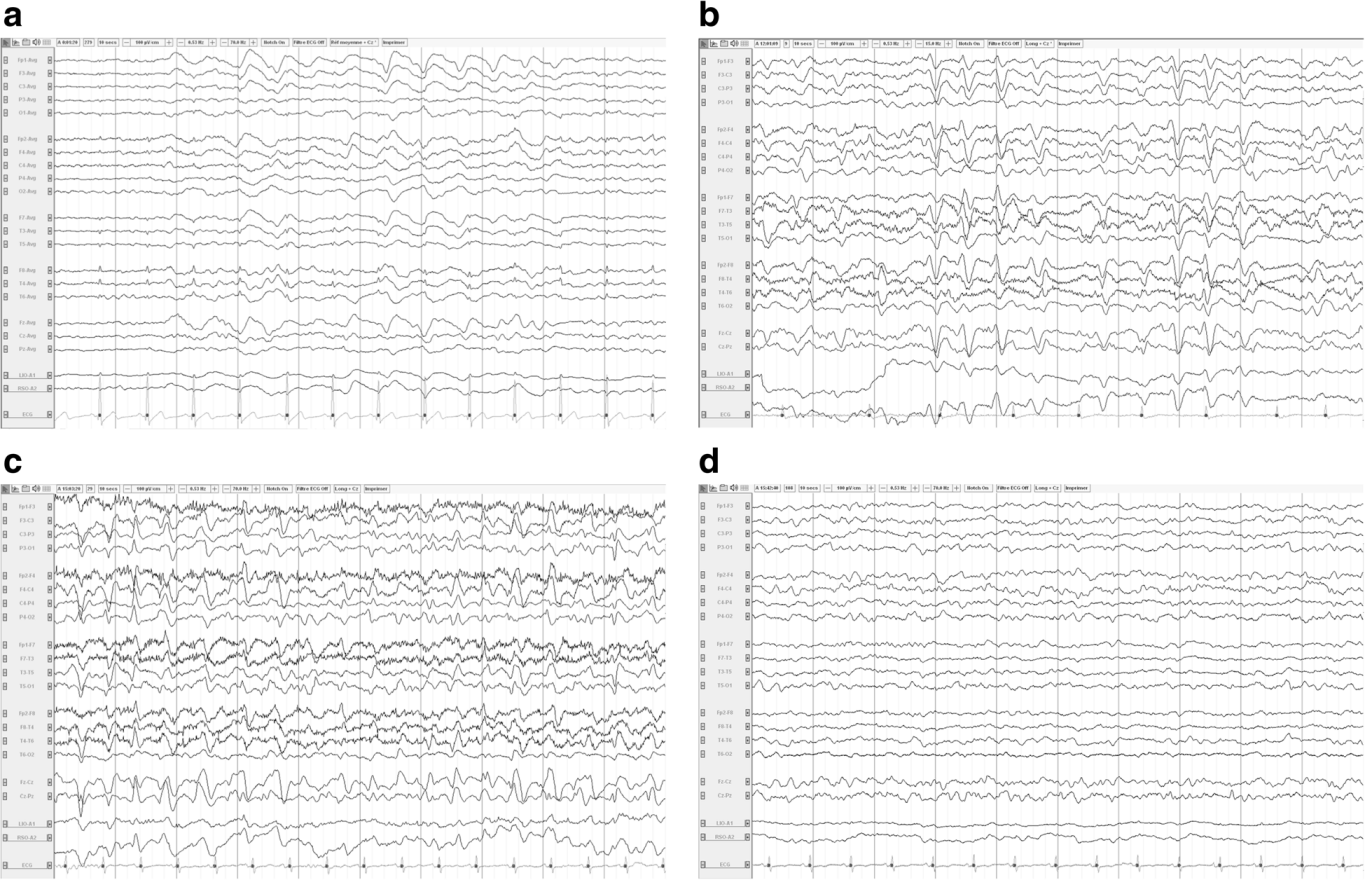
**Typical electroencephalogaphic (EEG) findings in patients with sepsis-associated brain dysfunction. a)** Frontal intermittent rhythmic delta activity (intermittent generalized rhythmic delta activity (GRDA), frontally predominant according to the ACNS Critical Care EEG terminology): burst of bilateral symmetrical and synchronous monomorphic delta (1.5 Hz in this case) activity predominating over the frontal regions. **b)** Triphasic waves (generalized periodic discharges (GPDs) with triphasic morphology according to the ACNS Critical Care EEG terminology): bilateral synchronous and symmetrical discharges occurring at a periodic interval (1.5 Hz); each discharge has three phases (negative–positive–negative), the second phase has the highest amplitude and each phase is longer in duration than the previous one. **c)** Nonconvulsive status epilepticus: presence of generalized periodic discharges at 2 to 2.5 Hz maximal over the frontal regions; discharges have a sharp wave morphology; independent sporadic sharp waves are present over the left posterior region. **d)** Moderate generalized slowing (same patient as in **c** after administration of IV levetiracetam): disappearance of generalized periodic discharges; mixed theta-delta background. ACNS, American Clinical Neurophysiology Society.

Similar information was provided by studies that included critically ill patients with a variety of diagnoses [27,28,31,32]. In a prospective series of 36 patients with systemic inflammatory response syndrome (SIRS) and signs of neurological dysfunction, encephalopathy was the most common manifestation, occurring in 75% of cases. The EEG background was abnormally slow in all 27 cases, although no additional details were provided [31]. The importance of background slowing was further investigated in a retrospective series of 154 hospitalized patients with altered mental status, including 64 (42%) with a non-cerebral infection [27]. The presence of background slowing with dominant theta-delta activity was associated with a poor outcome, although more severe slowing was not. The degree of slowing was associated with the severity of the encephalopathy, as assessed by the Glasgow Coma Scale (GCS) score, in a series of 105 patients with altered mental status and TWs [28]. Lack of EEG reactivity was reported in 28 of 154 patients admitted to a surgical ICU (100/154 had sepsis) and tended to be associated with worse outcome: only 3/28 patients with lack of EEG reactivity achieved a good outcome (moderate to no disability) at discharge, compared to 25/126 with EEG reactivity [32]. It should be noted that none of the aforementioned studies addressed the potential confounding effect of sedation on the EEG. In Figure 2, typical EEG findings in patients with sepsis-associated brain dysfunction are reported.

#### Periodic and rhythmic discharges

##### *Triphasic waves and periodic epileptiform discharges*

The presence of TWs was reported in three of the five case series of septic patients [18,20,21] and in two case series of critically ill patients [27,31]. Their incidence varied from 6% to 12% in patients with sepsis [18,20,21] and from 4% to 22% in the mixed population [27,31]. Two of these studies found that TWs were associated with a greater degree of cerebral dysfunction and greater mortality [18,27]. In a mixed population, TWs were more common in patients with liver or multi-organ failure [27] but they also occurred in septic patients without organ failure [18]. Other periodic epileptiform discharges, including GPEDs, PLEDs and BIPLEDs, were not described in any of the series that included only septic patients. In contrast, these epileptiform discharges were reported in a minority (17% to 29%) of patients undergoing continuous EEG monitoring in medical and surgical ICUs [30,32]. In one of these two studies, sepsis was a significant risk factor for periodic discharges whereas periodic discharges or seizures were independent predictors of poor outcome [30]; in the other study, only periodic discharges persisting more than 24 hours were associated with poor outcome [32]. The occurrence of periodic discharges in sepsis was further demonstrated in a large case-control study of 200 patients with GPEDs and 200 controls matched for age and alteration of consciousness [24]; the primary diagnosis was sepsis in 70/200 (35%) patients and the presence of GPEDs was associated with a longer ICU stay. Importantly, in three of these studies [24,30,32], TWs were classified as GPEDs.

##### *Frontal intermittent rhythmic delta activity*

FIRDA was described in one case series of patients with sepsis, occurring in 9/62 cases who also had predominant background delta activity [18]. Its occurrence in other EEG grades was not reported and it is thus impossible to make any conclusions about its significance. In another series, FIRDA was reported in 26 of 154 (18%) hospitalized patients with altered mental status and was associated with a better outcome [27].

#### Electrographic seizures

Electrographic seizures were described in only one of five case series using intermittent EEG recordings in septic patients [21]; in this study, they occurred in 13/43 (30%) patients. In contrast, studies in critically ill patients using continuous EEG monitoring found a 10% to 16% prevalence of electrographic seizures, including electrographic status epilepticus [24,30,32]. In one study, sepsis was an independent risk factor for developing electrographic seizures [30] and, in the other, all cases of electrographic status epilepticus occurred in septic patients [32]. In both series [30,32], seizures were independent predictors of poor outcome.

### Quantitative EEG analysis

Quantitative EEG was performed in three studies [16,19,26] (Table 3). A case-control study comparing 43 patients with community-acquired pneumonia to 30 controls reported a transient but significant decrease in the peak frequency and the relative power content of the alpha band, coupled with an increase in the relative power content of theta and beta bands [16]. In a study comparing linear and non-linear quantitative EEG measures, the authors found that both the spectral EEG ratio (the power content in the alpha and beta bands divided by the power content in the delta and theta bands) and the Kaplan z-score (a measure of non-linearity) were lower in patients with sepsis compared to normal values [19]. Only the Kaplan z-score was associated with sepsis severity, but it did not predict outcome.

**Table 3:** **Findings of quantitative EEG analysis in patients with sepsis**

	**Ref**	**Type of quantitative analysis**	**Findings**	**Diagnostic and prognostic value of the abnormalities**
**Case-control study (patients with systemic infection versus controls)**				
	16	Spectral analysis (Fast Fourier Transform)	Lower relative power in the alpha band; higher relative power in the theta and beta band; lower alpha peak frequency; higher theta peak frequency	NA
**Case series of patients with sepsis**				
	19	Spectral analysis (Fast Fourier Transform); nonlinear analysis (Kaplan z-score)	Lower spectral EEG ratio; higher Kaplan z-score	Kaplan z-score correlated negatively with the APACHE II score.
**Case series or case-control study that included patients with and without sepsis**				
	26	Burst suppression index (provided from a BIS monitor)	Burst suppression occurred in 49 of 125 patients (39%)	Diagnostic value of the presence of burst and suppression for sepsis (Sen = 0.42; Spe = 0.66; PLR = 1.2; NLR = 0.9). Burst suppression with higher six-month mortality (hazard ratio = 2.04, *P* = 0.02).

APACHE, Acute Physiology and Chronic Health Evaluation; BIS, bispectral index; EEG, electroencephalogram; NA, not applicable; NLR, negative likelihood ratio; PLR, positive likelihood ratio; Sen, sensitivity; Spe, specificity.

Finally, in one study, 49 out of 125 (39%) sedated critically ill patients had periods of EEG suppression, as assessed by the burst-suppression ratio of the BIS [26]. The presence of a burst-suppression pattern was not more common in patients with sepsis but was associated with an increased risk of mortality at six months.

### Evoked potentials

Five studies examined SSEPs [17,25], AEPs [22,29] or visual EPs (VEPs) [16] (Table 4). In both SSEP studies, peak latencies were significantly prolonged in patients with sepsis compared to controls [17,25]. Cortical responses and, in particular, the late response N70 generated by the somatosensory cortex ipsilateral to the stimulated median nerve and the N20-N70 latency (both dependent on intracortical conduction), were more frequently prolonged and to a greater extent. The delay was related to the severity of sepsis but unaffected by sedation. The relationship of SSEP prolongation to outcome was not assessed.

**Table 4:** **Abnormalities in evoked potentials in patients with sepsis**

	**Ref**	**Type of evoked potential**	**Alterations in the evoked potential**	**Rate (%) or findings**	**Other findings**
**Case–control study (patients with systemic infection versus controls)**					
	16	VEP	VEP N1-P1	no difference	
**Case series of patients with sepsis**					
	17	SSEP, including long-latency responses	N9, N20 and N70 peak latency; N13–N20 and N20–N70 peak-peak latency	Prolonged in 57%, 47%, 94%, 34% and 84% of patients, respectively	SSEP peak latencies correlated with the APACHE III score and were the same in sedated and non-sedated patients.
	22	AEP	I-V interwave latency	Prolonged in 4/6 (67%)	
**Case series or case–control study that included patients with and without sepsis**					
	29	AEP	15% decrease of the AAI	80% in sepsis patients	The median AAI of patients with SAE, 58 (range of 40 to 70) whereas it in ones without, 70 (55 to 90).
	25	SSEP, including long-latency responses	N9, N20 and N70 peak latency; N13–N20 and N20–N70 peak-peak latency	Prolonged in patients with sepsis compared to controls	The delay of N70 peak latencies correlated with the APACHE III score. Peak latencies were not different in sedated and non-sedated patients.

AAI, A-line Autoregression Index; ABSR, auditory brain stem response; AEP, auditory evoked potentials; APACHE, Acute Physiology and Chronic Health Evaluation; EEG, electroencephalogram; NA, not applicable; SAE, sepsis-associated encephalopathy; SSEP, somatosensory evoked potentials; VEP, visually evoked potentials.

AEPs were impaired in septic patients in two studies [22,29]. A small series reported that the I-V inter-wave latency (measuring conduction between the cochlear nerve and the rostral brainstem) was prolonged in 4/6 patients [22]. A larger study, using a quantitative index reflecting both the amplitude and latency of middle-latency AEP, thought to reflect activation of the auditory cortex, found a significant difference between patients who developed sepsis after surgery compared to those who did not, with sepsis patients having a lower index, suggesting reduced cortical activation [29]. The difference tended to be more pronounced in patients with SAE but this was not statistically significant.

In the only study reporting on VEPs, the amplitude of short-latency VEP was not affected by sepsis, although the hemodynamic response to stimulation was altered [16].

## Discussion

The objective of this systematic review was to summarize the available evidence concerning the utility of EEG and EP in the management of SAE/SABD. Despite more than 20 years of research, the role of these monitoring systems in SAE/SABD diagnosis and management remains unclear. Most of the available studies have important limitations, mainly related to small sample sizes, heterogeneity of the patient populations, retrospective study designs and lack of adequate controls (healthy individuals or patients with sepsis but no signs of encephalopathy). Many studies have also had clear selection biases, that is, they included mostly or exclusively patients with altered mental status and/or clinical seizures. This is especially true for the two studies that used continuous EEG monitoring [30,32]. Also, EEG and EPs assessment was not the primary aim in four studies and this may further limit the interpretation of these data.

Finally, the EEG definitions for seizures, background patterns and other electroencephalographic abnormalities (that is, GPEDs or PLEDs) have been only recently characterized [15] so that comparison of data coming from different studies and reproducibility of EEG interpretation are largely limited and biased.

Notwithstanding these limitations, we can conclude that EEG is a sensitive tool for detection and diagnosis of SAE/SABD. A slowing of the normal alpha rhythm with appearance of theta activity occurs in patients with no evidence of encephalopathy or with mild to moderate encephalopathy (confusion, delirium) and reflects cortical dysfunction. More severe states of altered consciousness (stupor and coma) are associated with increased slowing and the occurrence of delta activity, TWs and the more malignant burst-suppression patterns that indicate impaired function of deeper brain structures, such as the basal ganglia and the diencephalon.

Two prospective studies included a control group (patients with sepsis but no signs of SAE/SABD) and used similar but not identical EEG scales [18,20]. These studies both reported a high prevalence (>80%) of background abnormalities. One of the studies found an association between the severity of EEG abnormalities and the presence and severity of SAE/SABD [18]. This association was confirmed in a retrospective study of patients with encephalopathy that included patients with sepsis [27] but not in another study [20]. These conflicting results may be related to the different criteria used to define encephalopathy. In one study [18], the authors used a detailed mental state examination and graded the severity of encephalopathy (absent, mild or severe), whereas in the other [20], the authors relied on the Confusion Assessment Method for the ICU (CAM-ICU), which dichotomizes patients according to the presence or absence of delirium [33] and may not be sensitive enough to detect subtle forms of delirium [34]. This discrepancy highlights the lack of an agreed definition for SAE/SABD making it difficult to compare the accuracy of neurophysiological tests for diagnosis of SAE/SABD. This also suggests that the EEG might be more sensitive than simplified bedside cognitive scores to detect mild SAE/SBD, as was shown in other forms of encephalopathy [35]. Moreover, as an altered EEG always characterizes encephalopathy, the current criteria for SAE/SBD should also incorporate EEG findings in its definition.

Periodic and rhythmic discharges have been described in patients with sepsis but apart from TWs, which were associated with more severe encephalopathy and worse outcome, none has been specifically studied in patients with sepsis. Although the presence of periodic and rhythmic discharges was associated with a poor outcome in several series of critically ill patients, there is insufficient evidence to be able to draw conclusions on the prognostic significance of periodic and rhythmic discharges in sepsis. A similar conclusion can be reached for non-convulsive seizures, due to lack of specific evidence in the sepsis population.

It should also be noted that the study of periodic and rhythmic discharges used to be hampered by lack of homogeneity in definition. Terms such as TWs, which have received most attention in patients with SAE/SABD, are used inconsistently among neurophysiologists [36]. The field of critical care EEG has evolved tremendously over the last two decades and some recently described patterns [37,38] were not reported in older studies. Thus, the use of strict definitions for periodic and rhythmic patterns should be considered in future prospective trials and the impact of such EEG abnormalities in the setting of SAE further evaluated. Similarly, seizures are often difficult to discriminate from abnormal background and periodic or rhythmic discharges in patients with encephalopathy, leading to variable identification and poor inter-rater agreement [39]. Further studies should make use of standardized EEG terminology [15,40,41], which has been shown to have an excellent inter-observer reliability [37,38], to ensure consistent reporting and interpretation, and enhance generalizability and comparison of findings.

The EEG findings can be largely affected by the use of sedative drugs, although this phenomenon has not been well studied in the ICU setting. The typical EEG change due to deep sedation is a concomitant increase in slow (<1Hz) and alpha activity [42-44], a pattern that is not commonly observed in metabolic and septic encephalopathy. The occurrence of periodic or rhythmic patterns, or of an EEG background consisting of theta and delta waves without superimposed alpha activity, is more likely to indicate SAE in sedated patients with sepsis than the effects of sedation. Similarly, the so-called ‘malignant patterns’ (suppression-burst and electrical cerebral inactivity) are unlikely to be secondary to the use of moderate sedative regimens, as proposed in most ICU patients. In the same line, metabolic encephalopathy from liver or kidney failure can complicate sepsis, adding to SAE and potentially complicating EEG interpretation. This point has not been addressed in sufficient detail in published studies.

No study has specifically and prospectively investigated the use of continuous EEG monitoring in patients with sepsis, and thus no recommendation can be made on any potential advantage in this population. From studies in more general ICU populations [30,32,45], it can be concluded that prolonged recordings (>24 or even >48 hours in comatose patients) are required to identify most seizures, as short term-studies might miss up to 40% to 60% of patients with ictal discharges. Seizures occur in 10% to 20% of critically ill patients and are more frequent in patients with sepsis. Up to 90% of these seizures are non-convulsive and will be missed if continuous EEG is not performed [39]. It is currently unclear how aggressively non-convulsive seizures should be treated. However, two studies in non-neurological ICUs have reported that seizures are associated with worse outcome [30,32]. There is also a wealth of evidence in animals and humans that non-convulsive seizures are associated with detrimental physiologic and hemodynamic effects [46]. Altogether, these findings suggest that non-convulsive seizures probably warrant detection and treatment, which can only be performed using continuous EEG monitoring. Finally, as the time between the diagnosis of sepsis and the electrophysiological assessment widely varied from one to eleven days in these studies, the use of a continuous EEG monitoring could also help in better understanding the time-course of EEG/EPs changes in these patients and whether these changes are correlated with new clinical events.

Quantitative EEG analysis has the potential to reduce the burden of EEG analysis compared to standard EEG and especially continuous EEG monitoring, by converting complex multi-channel recordings into simpler numerical outputs, which can be plotted and displayed at the bedside and interpreted by non-neurophysiologists. Currently available methods have reasonable sensitivity and specificity for seizure detection and for the identification of delayed ischemia after subarachnoid haemorrhage [47]. Currently available data suggest that some quantitative EEG measures are sensitive to the presence of brain dysfunction. There is insufficient evidence to recommend their use in the management of SAE/SABD, although the use of BIS and similar measures was recently suggested as an additional tool to monitor sedation in patients receiving neuromuscular blockers [48].

Two prospective case-control studies reported on EP alterations, with different results [16,17]. Compared to healthy controls, there was no difference in the amplitude of short-latency VEPs in patients with sepsis [16], whereas short-latency and long-latency SSEPs (N70) were significantly delayed in patients with sepsis [17]. This discrepancy might underscore the fact that cortico-cortical conduction is more sensitive to the effects of sepsis than conduction from deeper structures to the cortex. These EP alterations were proportional to the severity of sepsis and are not affected by sedation [17], which is an advantage compared to EEG readings. No data are available regarding the relationship of EP alterations to the presence of encephalopathy or the prognostic significance of these abnormalities.

Another uncertainty lies in the nature of the observed EEG/EP abnormalities. The pathophysiology of sepsis is complex and multifactorial [1,5]. Brain hypoperfusion and ischemia are major contributors to the encephalopathy, through systemic hypotension [22,49,50], loss of cerebral autoregulation [16,51,52] and microvascular dysfunction [7,53,54], but additional mechanisms, including functional perturbation of neurotransmission and neuronal function also occur. Moreover, the clinical picture can be further complicated by the occurrence of organ dysfunction and of electrolytic disorders or the use of potentially neurotoxic drugs, such as some antibiotics [55,56], which can all affect the EEG. These confounders have not been specifically assessed in studies on EEG and EPs in septic patients and may also have influenced final results.

Whether EEG/EP findings represent irreversible brain damage, temporary dysfunction or whether EEG abnormalities may contribute to brain injury in septic patients, remains unknown. This should be further investigated in prospective studies before one can conclude that these measures are merely a marker of severity, with only diagnostic and/or prognostic value, or can be used as a target for resuscitation and treatment. As discussed above, it is imperative that these prospective studies make use of validated EEG terminology to ensure their generalizability and reproducibility. Finally, EEG can be helpful to monitor the depth of sedation, to reveal particular and treatable features (such as seizures) or to indicate neuroimaging in the case of malignant patterns; however, whether EEG monitoring has a significant impact on the diagnostic and therapeutic algorithms used in septic patients with SAE should also be evaluated in large prospective studies.

## Conclusions

The limited available studies indicate that EEG abnormalities occur in a majority of patients with sepsis and are associated with the presence and severity of SAE/SABD. Some abnormalities, such as TWs, delta slowing and a suppression-burst pattern, may indicate a poorer prognosis but these observations need to be confirmed in larger prospective controlled studies. Given the high prevalence (10% to 20%) of non-convulsive seizures in patients with sepsis, EEG monitoring is probably indicated, especially in patients with severe or unexplained alteration of consciousness. Based on studies in general populations of critically ill patients, the recommended minimal duration of monitoring is 24 hours, and 48 hours in comatose patients. There is currently not enough evidence to recommend the use of quantitative EEG analysis and EPs but the available data are promising and suggest that these parameters should be studied further.

## Key messages

- The incidence of EEG abnormalities is high during sepsis.
- EEG abnormalities are related to the severity of encephalopathy; however, they can be present also in patients without clinical signs of encephalopathy.
- Evoked potentials are altered during sepsis.
- The association of EEG and EP abnormalities with outcome needs to be further evaluated.

## Abbreviations

AEP: auditory evoked potential
APACHE: Acute Physiology and Chronic Health Evaluation
BAEP: brainstem auditory evoked potential
BIPLEDs: bilateral independent lateralized epileptiform discharges
BIS: bispectral index
CNS: central nervous system
EEG: electroencephalogram
EP: evoked potential
FIRDA: frontal intermittent rhythmic delta activity
GCS: Glasgow Coma Scale
GPEDs: general periodic epileptiform discharges
PLEDs: periodic lateralized epileptiform discharges
PRISMA: Preferred Reporting Items for Systematic Reviews and Meta-Analyses
qEEG: quantitative electroencephalogram
SABD: sepsis-associated brain dysfunction
SAE: sepsis-associated encephalopathy
SIRS: systemic inflammatory response syndrome
SSEP: somatosensory evoked potential
TWs: triphasic waves
VEP: visually evoked potential
